# Dynamics-based data science in biology

**DOI:** 10.1093/nsr/nwab029

**Published:** 2021-02-12

**Authors:** Jifan Shi, Kazuyuki Aihara, Luonan Chen

**Affiliations:** International Research Center for Neurointelligence, The University of Tokyo Institutes for Advanced Study, The University of Tokyo, Japan; International Research Center for Neurointelligence, The University of Tokyo Institutes for Advanced Study, The University of Tokyo, Japan; Shanghai Institute of Biochemistry and Cell Biology, Center for Excellence in Molecular Cell Science, Chinese Academy of Sciences, China; Center for Excellence in Animal Evolution and Genetics, Chinese Academy of Sciences, China; School of Life Science and Technology, ShanghaiTech University, China; Key Laboratory of Systems Biology, Hangzhou Institute for Advanced Study, University of Chinese Academy of Sciences, Chinese Academy of Sciences, China

## Abstract

With the increasingly accumulated bio-data, dynamics-based data-science has been progressing as an efficient way to reveal mechanisms of dynamical biological processes. We review three applications on detecting the tipping-points of diseases, quantifying cell's potency, and predicting time-series, to show the importance of dynamics-based data-science.

Life science has long been a rich subject for research, and continues to develop at high speed. One of the major aims of life science is to study the mechanisms of various biological processes on the basis of biological big-data. Many statistics-based methods have been proposed to catch the essence by mining such data, including the popular category classification, variables regression, group clustering, statistical comparison, dimensionality reduction, and component analysis. However, these mainly elucidate static features or steady behavior of living organisms because of a lack of temporal data. A biological system is inherently dynamic, and with increasingly accumulated time-series data, there is a need for dynamics-based approaches based on physical and biological laws to reveal dynamic features or complex behavior of biological systems [1]. In this perspective, we review three dynamics-based data science approaches for studying dynamical bio-processes: namely, dynamical network biomarkers (DNB), landscapes of differentiation dynamics (LDD) and autoreservoir neural networks (ARNN). They are all data-driven or model-free approaches but based on the theoretical frameworks of nonlinear dynamics, that is, ordinary differential equations (ODE), partial differential equations (PDE) and artificial neural networks (NN), respectively. Figure 1A and B illustrates dynamical bio-processes and their omics data in biomedical fields, while Fig. 1C summarizes the three approaches of dynamics-based data science, which serve as typical examples for studying biological systems from a data-driven dynamical perspective.

- **Providing early warning signals of pre-diseases/tipping-points by bifurcation theory.** The rapid development of high-throughput technology is allowing measurement of omics data to become more precise and less time-consuming. Identifying the tipping-point/pre-disease state is an urgent task for individuals in precision and prevention medicine. The DNB theory [2] is a dynamics-based data-driven approach to quantify critical states [1] during disease progression, as shown in the first column of Fig. 1C. A so-called pre-disease state (between normal and disease states) just before the disease state is defined as the state just before the bifurcation point of the dynamic system (ODE), which can be detected only by data based on three statistical conditions: (1) average standard deviations of DNB-members drastically increase; (2) Pearson correlation coefficients (PCCs) between DNB-members drastically increase in absolute values; (3) PCCs between DNB- and non-DNB-members drastically decrease. The three conditions are generically derived by exploring the low-dimensional feature of the center manifold just before the bifurcation of a fixed point (equilibrium) attractor in a nonlinear dynamical system. DNB not only quantitatively detects the ‘Wei-Bing’/pre-disease state or predicts the imminent disease state for predictive medicine, but also provides early warning signals of other critical transitions, such as influenza outbreaks or even pandemics [2,3].
- **Quantifying cell potential landscape by diffusion map theory with divergence theorem.** Single-cell sequencing data provide us the opportunity to study heterogeneity of tissues and capture features of different stages in cell differentiation processes. Various methods have been proposed to construct the differentiation tree, identify cells’ pseudo-time trajectory of differentiation, and study the transition rate between different cell-states [4,5]. In contrast to the traditional distance-based and entropy-based methods, LDD describes the cell differentiation as a non-equilibrium system by considering birth and death of cells on the basis of a source-sink Fokker-Planck equation (PDE) [6,7], as shown in the second column of Fig. 1C. By exploring diffusion map theory and divergence theorem, LDD can numerically estimate the potential of each cell cluster only with scRNA-seq data. In particular, LDD can not only identify the stem cell cluster with the highest pluripotency but also construct Waddington's potential [8], without specific prior knowledge on stem cells and cell flow rates, which are required by the previous algorithms.
- **Predicting time-series from short-term high-dimensional data by delay embedding theory.** Predicting future values from a short-term time-series is a difficult task but a hot topic. To overcome the small sample size problem, spatiotemporal information transformation (STI) equations with nonlinear functions were derived based on the Takens’ delay embedding theory for a nonlinear dynamic system [9] and its generalization [10]. ARNN [11] utilizes the STI equations by taking advantage of the NN’s ability to approximate any complicated nonlinear function, from the short-term high-dimensional data, as described in the third column of Fig. 1C. The STI equations represent the essential dynamics from the data, which equivalently enlarge the sample size and thus make the prediction accurate. In particular, different from the traditional reservoir structure with external dynamics [12], ARNN adopts nonlinear dynamics of a target system to efficiently transform the high-dimension spatial data into the low-dimension temporal data of a target variable, that is, future values or prediction [11]. ARNN not only could reduce the consumption of computing resources, but also avoids overfitting caused by much fewer training parameters. ARNN has been shown to have superior performances in predicting short-term time-series, such as gene expressions, daily cardiovascular disease admissions, wind speed, sea-level pressure, etc.

**Figure 1.**
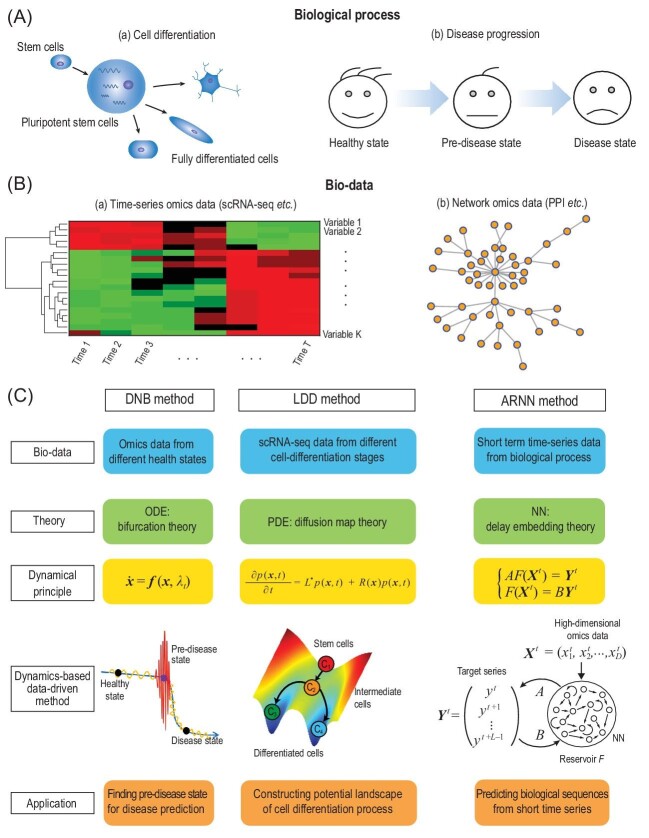
Dynamics-based data science approaches. (A) An illustration for a dynamic biological process, which usually includes a series of stages or states. (B) An illustration for omics-data extracted from experiments. (C) Three examples of dynamics-based data science approaches. The first column is the dynamic network biomarker (DNB) framework. DNB provides early warning signals for pre-disease/tipping-point detection from omics data of different health states, based on the bifurcation theory with ordinary differential equations (ODE). The second column is the landscape of a differentiation dynamics (LDD) framework, which distinguishes different cell types, provides pseudo-time trajectory for cell clusters (C_1_, C_2_, C_3_ and C_4_), and constructs a potential landscape for the cell differentiation process from single-cell RNA-sequencing (scRNA-seq) data during a cell differentiation process, based on the diffusion map theory of partial differential equations (PDE) named Fokker-Planck equations with source-sink terms. The third column is the autoreservoir neural network (ARNN) framework, which is able to predict short time-series from high-dimensional data by spatiotemporal information transformation (STI) and also significantly save computing resources, based on the delay-embedding and generalized embedding theorems of dynamic systems.

The efficiency of dynamical-based data science approaches on biological data has been demonstrated by the three methods above, which all show strong power in solving biological questions and are complementary to traditional statistics-based data science approaches. In addition, dynamical-based data science approaches can be applied to dynamical causality detection by exploring continuity of the cross mapping function between the observed variables. From a methodological viewpoint, we can summarize how to build a dynamics-based data-driven approach for studying biological dynamics as follows.

- It generally starts from basic laws which a biological process obeys, e.g. proper dynamical equations for describing its time evolution. Note that how to narrow down to an appropriate dynamical model, e.g. ODE, PDE or NN, depends on the specific situation or prior information of the problem under study.
- Then, we need to derive the generic or essential statistic features, that can be characterized by data, from such dynamical equations or models.
- With such features characterized by data, we can quantify various dynamical processes of biological systems based only on the measured data in a fully model-free manner.

Taken together, we conclude that the principles and advantages of dynamics-based data-driven approaches are explicable, quantifiable and generalizable. ‘Explicable’ indicates that every term in the dynamics-based data-driven approaches has its physical or biological sense. ‘Quantifiable’ ensures that the system can be measured by objective criteria and is comparable by quantitative indicators. ‘Generalizable’ says that the method can be improved by adding new factors or be generalized to other systems by proper modifications. In particular, dynamics-based data science approaches exploit the essential features of dynamical systems in terms of data, e.g. strong fluctuations near a bifurcation point, low-dimensionality of a center manifold or an attractor, and phase-space reconstruction from a single variable by delay embedding theorem, and thus are able to provide different or additional information to the traditional approaches, i.e. statistics-based data science approaches. We believe that dynamical-based data science approaches will play an important role in systematic research in biology and medicine in future.
